# The short fiber knobs of human adenovirus in species F elicit cross-neutralizing antibody responses

**DOI:** 10.1016/j.heliyon.2024.e35783

**Published:** 2024-08-03

**Authors:** Zhenwei Liu, Guolu Tang, Yinghui Peng, Jixian Lan, Yuting Xian, Xingui Tian, Dehui Chen

**Affiliations:** a Department of Pediatrics, The First Affiliated Hospital of Guangzhou Medical University, Guangzhou, China; b State Key Laboratory of Respiratory Disease, Guangdong-Hong Kong-Macao Joint Laboratory of Respiratory Infectious Disease, Guangzhou Medical University, Guangzhou, China; c State Key Laboratory of Respiratory Disease, National Clinical Research Center for Respiratory Disease, Guangzhou Institute of Respiratory Health, The First Affiliated Hospital of Guangzhou Medical University, Guangzhou Medical University, Guangzhou, China; d Guangdong Sanmai Biotechnology Co., Ltd, Guangzhou, China

**Keywords:** Human adenovirus type 40 and 41, Short fiber knob, Cross-neutralization

## Abstract

Human adenovirus (HAdV) type 40 in species F (HAdV-F40) and HAdV-F41 represent the third most prevalent causative agents of non-bacterial acute gastroenteritis in infants and young children, following norovirus and rotavirus. Despite their significant contribution to global child morbidity, vaccines to preemptively combat these viruses remain elusive. In this study, we investigate the potential for cross-neutralization between HAdV-F40 and HAdV-F41 using immune sera with the short fiber knob (SFK). We implemented a series of assays to evaluate the responses, including enzyme-linked immunosorbent, micro-neutralization, immunofluorescence, and quantitative polymerase chain reaction. Our results demonstrate that immune sera with HAdV-F40 SFK or HAdV-F41 SFK could effectively neutralize both HAdV-F40 and HAdV-F41, indicating a mutual cross-neutralizing effect. Notably, the immune sera with HAdV-F40 SFK demonstrated a stronger neutralization effect, suggesting the potential to develop a subunit vaccine that can simultaneously counteract both viruses. These findings underscore the potential of SFK immunization in evoking a cross-neutralizing antibody response between HAdV-F40 and HAdV-F41. This suggests a promising avenue for developing subunit vaccines against HAdV-F40 and HAdV-F41 and provides a novel perspective on the potential of neutralizing antibodies to protect against these two types of HAdV.

## 1 Introduction

First discovered over seven decades ago, human adenoviruses (HAdVs) persist as significant health concerns, especially within populations with naive or compromised immune responses [1]. Their pathogenic manifestations span a range of clinical presentations, such as acute respiratory disease, cystitis, gastroenteritis, and keratitis [[2], [3], [4], [5]]. Driven by advancements in genomic studies, there has been the identification and characterization of over 100 HAdV types as documented by (http://hadvwg.gmu.edu/), categorized into seven distinct species (A–G) [3]. HAdV type 40 in species F (HAdV-F40) and HAdV-F41 are predominantly responsible for non-bacterial acute gastroenteritis in younger populations, with only norovirus and rotavirus being more prevalent [[6], [7], [8]]. HAdV-F40 and HAdV-F41 are significant causes of diarrhea and diarrhea-associated mortality in young children. Diarrhea leads to approximately 0.5 million deaths per year in children younger than 5 years worldwide, underscoring the importance of identifying an effective immunization strategy. Contemporary research underscores the prevalence of these enteric adenoviruses, reporting a detection rate of 5.18 % in pediatric diarrhea patients. Their global prevalence oscillates between 1.2 % and 15 % [[9], [10], [11], [12]]. In certain regions such as China, these viruses have been implicated in outbreaks of severe pediatric acute hepatitis, underscoring their pathogenic potential [13,14]. Emerging data also posits a possible synergistic effect between HAdV-F41 and SARS-CoV-2 in inducing acute hepatitis in pediatric cohorts, with the direst cases necessitating liver transplantation [15]. Predominantly, children below the age of 5 are the primary recipients of HAdV infections, where HAdV-F40 and HAdV-F41 dominate. Although our comprehension of these viruses has expanded considerably, the formulation of an efficacious vaccine eludes the scientific community, and the absence of potent antiviral therapeutics exacerbates the challenge.

Structurally, HAdVs are non-enveloped viruses approximately 90–100 nm in diameter, featuring an icosahedral nucleocapsid formed by three major and four minor structural proteins [16,17]. Central among these is the fiber protein, segmented into a tail, a shaft, and a knob. The fiber knob, despite being subdominant, elicits neutralizing antibodies (NAbs) in vaccinated and naturally exposed individuals [18,19]. A distinctive trait of HAdV-F40 and HAdV-F41 is their unique fiber composition, featuring both long and short fibers, each emerging from the penton base at each vertex of the virus. This configuration marks their specialized mode of host interaction. Specifically, each virion displays a pentameric base at the vertices of its icosahedral structure, from which two types of trimeric fiber proteins extend: the long fiber knob (LFK) and the short fiber knob (SFK). The SFK protrudes directly upward from the penton base, while the LFK projects at an angle, providing diverse binding capabilities that facilitate viral attachment to host cells. The distal knob domains of these fibers engage specific cellular receptors, crucially influencing the virus's ability to infect host cells [20,21]. Importantly, it is the SFK plays a significant role during the course of viral infection [22]. While recent progress has begun to illuminate the intricacies of adenoviral structures, the distinctive nature of HAdV-F40 SFK and HAdV-F41 SFK invites deeper investigation [23,24]. Prior to our focus on the cross-neutralizing potential of antigenic epitopes, a comprehensive examination of the knob structure in these HAdVs and its implications on virus-host interactions is critical. Our previous work identified cross-neutralizing and cross-binding antibodies targeting the species B fiber knob [25,26]. These discoveries significantly enrich our understanding of the antibody response to these viruses. Consequently, unraveling the structure and function of the fiber knobs, both SFK and LFK, can provide critical insights into developing new antiviral strategies against adenoviral infections.

In this study, we utilized HAdV-F40 SFK and HAdV-F41 SFK, expressed in an Escherichia coli (*E. coli*) system, as immunogens for mouse immunization. We then assessed the immune sera for cross-neutralizing activity against HAdV-F40 and HAdV-F41. Our results indicate that both HAdV-F40 SFK and HAdV-F41 SFK can induce cross-neutralizing antibody responses, suggesting their potential as subunit vaccine candidates against these viruses.

## 2 Materials and methods

### 2.1 Animals, cells and viruses

Specific pathogen-free (SPF) BALB/c mice, aged four weeks, were acquired from the Guangdong Medical Laboratory Animal Center (Foshan, Guangdong, China). These mice were maintained in individually ventilated cages in an SPF environment. Ambient conditions were regulated at 25 °C and a relative humidity of 50–65 %. All mice were given free access to sterilized food and water.

The HEK-293 cells used in this study were obtained from the American Type Culture Collection (ATCC). These cells were cultivated in a culture medium supplemented with 10 % heat-inactivated fetal bovine serum (Gibco BRL, Grand Island, USA). Antibiotic supplementation included 100 U/ml penicillin and 100 μg/ml streptomycin (Gibco BRL, Grand Island, USA). The cells were incubated at 37 °C in a 5 % CO_2_ atmosphere.

The specific HAdVs employed in this study are detailed in Table 1. Each HAdV type was cultured and titrated utilizing HEK-293 cells. Viral titers were determined using the 50 % tissue culture infectious dose (TCID_50_) method, as described by the Reed-Muench methodology. This determination was predicated on the cytopathic effects (CPEs) [27]. All viral strains referenced in Table 1 were amplified within our controlled laboratory environment.

**Table 1:** The viruses used in this study.

Virus	Strain	Reference
HAdV-F40	Dugan	ATCC®VR-931^TM^
HAdV-F41	Tak	ATCC®VR-930^TM^

### 2.2 Expression and purification of SFKs

Initiating with the synthesis of codon-optimized genes for HAdV-F40 SFK and HAdV-F41 SFK, these were then subcloned into the pQE-30 vector (Qiagen, Hilden, Germany). Following sequence validation, the resultant plasmid was transformed into *E. coli* M15 cells, which were maintained in-house. These cells were cultivated in LB medium (10 g/L tryptone, 5 g/L yeast extract, 10 g/L NaCl, and distilled water, autoclaved) supplemented with ampicillin (100 mg/ml) and kanamycin (50 mg/ml) (TransGen Biotech, Beijing, China), and incubated at 37 °C with agitation at 250 rpm overnight. The culture was continued under similar conditions until its optical density (OD) value at 600 nm reached a value between 0.5 and 0.7. Subsequently, isopropyl β-d-1-thiogalactopyranoside (TransGen Biotech, Beijing, China) was added to a final concentration of 0.5 mM and the culture was incubated at 18 °C with 250 rpm shaking overnight. Bacterial cells were harvested by centrifugation at 10,000 *g* for 20 min at 4 °C. The resulting pellet was resuspended in non-denaturing lysis buffer (0.05 M NaH_2_PO_4_·2H_2_O, 0.3 M NaCl, ddH_2_O, pH 8.0) supplemented with 1 mg/ml lysozyme and *ProteinSafe*® Protease Inhibitor Cocktail, EDTA-free (TransGen Biotech, Beijing, China) at a 1:100 dilution. This mixture was incubated on ice for 30 min, followed by sonication as per standard protocols (The ultrasonic power is set to Max. Each round of sonication lasts for 2 s, with 2 s intervals between rounds. There are a total of 8 rounds of sonication. The pattern follows: 3 rounds - pause - 3 rounds - pause - 2 rounds - pause - 2 rounds of sonication, with a pause time of 5 min in between.), and then centrifuged at 10,000 *g* for 30 min at 4 °C. The clear supernatant was subsequently filtered through a 0.22 μm membrane.

For protein purification, we utilized 2 ml of BeyoGold™ His-tag Purification Resin (Beyotime Biotechnology, Shanghai, China) packed into a 6 ml centrifuge column. Following the gravity-driven removal of storage solution, the protein was loaded onto the column, which was then sequentially washed and eluted using non-denaturing lysate (0.05 M NaH_2_PO_4_·2H_2_O, 0.3 M NaCl, ddH_2_O, pH 8.0), washing solution (0.05 M NaH_2_PO_4_·2H_2_O, 0.3 M NaCl, 0.02 M imidazole, ddH_2_O, pH 8.0), and eluent (0.05 M NaH_2_PO_4_·2H_2_O, 0.3 M NaCl, 0.04 M imidazole, ddH_2_O, pH 8.0), adhering to the manufacturer's instructions.

The purified SFK was then concentrated and desalted with the Amicon® Ultra-15 centrifuge filter (Millipore, Bedford, USA) by repeated cycles of centrifugation and resuspension in phosphate-buffered saline (PBS) (Thermo Fisher Scientific, Waltham, USA). The final protein concentration was determined using the NanoDrop™ One spectrophotometer (Thermo Fisher Scientific, Waltham, USA).

### 2.3 Sodium dodecyl sulfate-polyacrylamide gel electrophoresis (SDS-PAGE) analysis of SFKs

We used the SDS-PAGE denaturing polyacrylamide gel rapid preparation kit (Sagon Biotech, Shanghai, China) to prepare a 10 % resolving gel and a 5 % stacking gel. Two aliquots of SFK solution were prepared. Subsequently, 6 × Protein Loading Buffer (TransGen Biotech, Beijing, China) was added to each aliquot. The samples were treated differently: one was incubated at room temperature for 10 min while the other was subjected to boiling for an equivalent duration. This experimentation provided insights into the protein behavior under contrasting thermal conditions.

Electrophoresis was conducted using 1 × Tris-glycine SDS buffer (Sagon Biotech, Shanghai, China). Once bromophenol blue approached the bottom edge of the gel, electrophoresis was stopped. The separation gel was then rinsed with ddH₂O and stained with BeyoBlue™ Coomassie Blue Super-Fast Staining Solution (Beyotime Biotechnology, Shanghai, China) at room temperature on a decolorization shaker for 60 min. The post-stain solution was discarded, and the gel was rinsed again with ddH₂O. Decolorization was then performed for 60 min at room temperature. Finally, after discarding the decolorizing solution, the gel was left to undergo overnight decolorization in ddH₂O at 4 °C. This SDS-PAGE methodology allowed for the evaluation of the purity and structural integrity of SFKs under different conditions.

### 2.4 Mouse immunization

Four-week-old female BALB/c mice were allocated randomly into three distinct groups (n = 5 mice/group). Each group received intraperitoneal injections at bi-weekly intervals with the following treatments: purified HAdV-F40 SFK, purified HAdV-F41 SFK, or PBS as a blank control. Prior to immunization, blood was drawn from all subjects via tail vein incision, and this initial serum was designated as the negative control for subsequent enzyme-linked immunosorbent assay (ELISA) evaluation. On day 0, mice designated for SFK treatments were injected intraperitoneally with 200 μl of a solution comprising 50 μg of SFK and Freund's complete adjuvant (FCA) (SFK solution: FCA, volume ratio = 1:1). Mice in the blank control group received an equivalent volume of PBS in lieu of the SFK solution. Subsequent booster injections were given on days 14 and 28 using the initial immunization dose but paired with Freund's incomplete adjuvant (FIA) (SFK solution: FIA, volume ratio = 1:1). Blood samples were procured on days 14, 28, and 42. After the final immunization, all mice were euthanized, and their blood was subjected to centrifugation at 5000*g* for 5 min at 4 °C to separate serum efficiently. Resultant serum samples were extracted and preserved at −80 °C pending further analysis.

### 2.5 Serum titers by ELISA

Serum titers of each mouse group were determined using ELISA, with serum from the PBS-immunized group serving as a negative control. The SFK was diluted to a concentration of 2 μg/ml using 1 × ELISA coating buffer (pH 9.6) (Solarbio Science & Technology, Beijing, China) with an initial concentration of 0.5 mg/ml. The diluted SFK was used to coat F96 MAXISORP NUNC-IMMUNO PLATE (Thermo Fisher Scientific, Waltham, USA) by dispensing 100 μl into each well and incubating overnight at 4 °C. The wells were then emptied and washed once with 0.05 % PBST, 200 μl/well, and tapped dry. Thereafter, blocking buffer containing BSA (Sagon Biotech, Shanghai, China) was added at 200 μl/well and incubated at 25 °C for 2 h. Each serum sample was initially diluted to a starting dilution of 1:1000 for the baseline measurement. Subsequent serial dilutions were made at 1:10^4^ and 1:10^5^ to assess the maximum extent of the immunological response. Endpoint titers were defined as the highest dilution at which specific antibody binding was detectable above the background level. The buffer was then discarded, wells tapped dry, and diluted mouse serum added. Three wells per serum were used, and three blank controls received the ELISA universal antibody diluent at 100 μl/well. Plates were incubated at 37 °C for 1 h. The wells were subsequently washed six times with 0.05 % PBST (Solarbio Science & Technology, Beijing, China), 300 μl/well, and tapped dry. Next, *ProteinFind*® Goat Anti-Mouse IgG (H + L) (TransGen Biotech, Beijing, China) was diluted with ELISA universal antibody diluent and then added to each well. HRP Conjugate was subsequently added at a dilution of 1:5000 (TransGen Biotech, Beijing, China) at 100 μl/well, followed by incubation. Subsequently, Super TMB ELISA Substrate (TransGen Biotech, Beijing, China) was added at 100 μl/well, incubated in the dark at 25 °C for 3 min, and then the reaction was stopped by adding ELISA stop solution (Sagon Biotech, Shanghai, China) at 100 μl/well. OD values were measured at 450 nm and 630 nm within 30 min, with the calibrated OD calculated by subtracting the 630 nm reading from the 450 nm reading. Average OD values were computed for each dilution of each mouse group. The titer, considered as the highest dilution with a ratio greater than 2.1, represented the serum titer for that mouse group.

### 2.6 Micro-neutralization assay (MNA)

Serum samples underwent serial dilutions, yielding ratios of 1:18, 1:72, 1:288, 1:1152, and 1:4608. Both HAdV-F40 and HAdV-F41 were cultured in HEK-293 cells. Serum in the 96-well plate was serially diluted two-fold using DMEM/F-12 (Gibco BRL, Grand Island, USA), starting from a 1:9 dilution and was then combined with a viral suspension containing 200 TCID_50_/0.1 ml. This mixture was incubated at 37 °C in a 5 % CO_2_ environment for 1 h. Subsequently, the virus-serum mixture, at a volume of 100 μL per well, was inoculated in triplicate onto the monolayer of HEK-293 cells in the 96-well plate (at 80 % confluency). After incubation at 37 °C with 5 % CO_2_ for 2 h, the supernatant in each well was removed. Then, 100 μL of DMEM/F-12 (Gibco BRL, Grand Island, USA) was added to each well. The cells were then incubated at 37 °C with 5 % CO_2_ for 3 days post-inoculation, to observe and discriminate between complete and partial CPEs. Each assay incorporated control wells of uninfected cells and virus only. The highest serum dilution that completely inhibited CPEs in 50 % of the wells was defined as the neutralizing titer.

### 2.7 Immunofluorescence assay (IFA)

Following the evaluation of CPEs, the HEK-293 cells were prepared for immunofluorescence analysis. After removing the supernatant from the wells, cells were fixed overnight with 100 μl/well of a fixative solution (Beyotime Biotechnology, Shanghai, China) at 4 °C. Cells were subsequently washed (Beyotime Biotechnology, Shanghai, China) three times and then blocked using QuickBlock™ Immunostaining Blocking Buffer (Beyotime Biotechnology, Shanghai, China).

Post-blocking, the buffer was replaced with Mouse Anti-HAdV Monoclonal Antibodies (Hecin Scientific, Guangzhou, China) specific to either HAdV-F40 or HAdV-F41, which had been diluted in QuickBlock™ Immunostaining Primary Antibody Dilution Buffer (Beyotime Biotechnology, Shanghai, China), and incubated for 1 h at 25 °C. After this incubation, the cells underwent five washing steps. Subsequently, *ProteinFind*® Goat Anti-Mouse IgG (H + L) (TransGen Biotech, Beijing, China) was added, and diluted to a 1:5000 ratio with QuickBlock™ Immunofluorescence Secondary Antibody Dilution Buffer (Beyotime Biotechnology, Shanghai, China), at 100 μl per well and the plates were then placed on a shaker at 200 rpm and agitated at 25 °C for 1 h to ensure effective mixing and optimal interaction. Upon completion of this incubation, the cells were washed (Beyotime Biotechnology, Shanghai, China) another five times, followed by the addition of 100 μl/well of PBS. Immunofluorescent images were captured using a DM IL LED Microscope (Leica, Wetzlar, Germany) and a fluorescence microscope (Leica, Wetzlar, Germany). As a control, uninfected cells were also included. Through this IFA methodology, we were able to elucidate the neutralizing capability of antibodies in response to HAdV-F40 SFK and HAdV-F41 SFK.

### 2.8 Statistical analysis

Statistical differences were analyzed using one-way analysis of variance (ANOVA). The Brown-Forsythe test validated the assumption of homogeneity of variances. All analyses were conducted using GraphPad Prism software (Version 9.5.1, GraphPad Software Inc.). Differences were considered statistically significant when the *p*-value was less than 0.05.

## 3 Results

### 3.1 Expression and characterization of HAdV-F40 SFK and HAdV-F41 SFK

**Fig. 1:**
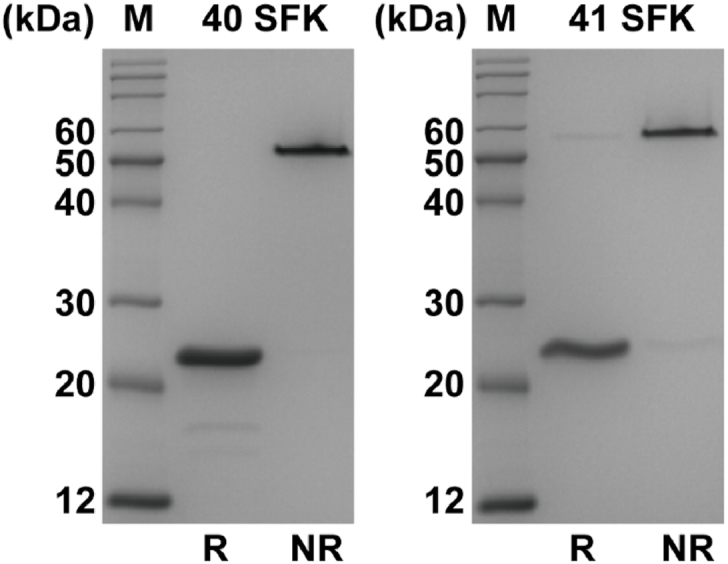
SDS-PAGE examination of purified HAdV-F40 SFK and purified HAdV-F41 SFK. An analysis of purified HAdV-F40 SFK and purified HAdV-F41 SFK, preheated (labeled as R) and non-preheated (labeled as NR), was undertaken using SDS-PAGE under denaturing and reducing conditions.

After denaturation, HAdV-F40 SFK and HAdV-F41 SFK displayed bands aligned with their anticipated size. Preheating resulted in bands consistent with the molecular weight expected for monomeric proteins. Without preheating, the purified SFKs manifested in polymeric forms (Fig. 1). The obtained SFKs exhibited a purity exceeding 90 %.

### 3.2 HAdV-F40 SFK and HAdV-F41 SFK elicited humoral immune responses

**Fig. 2:**
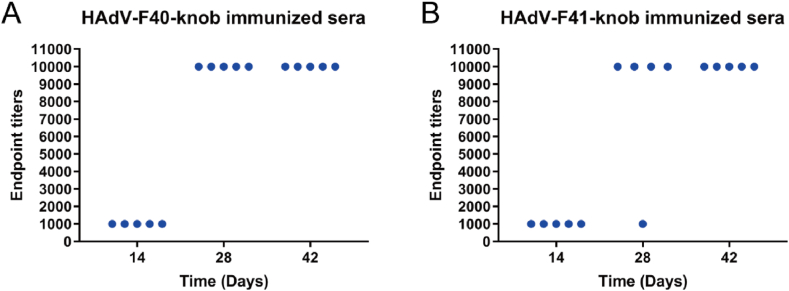
Evolution of the immune sera response over time post-immunization. Endpoint titers of immune sera are displayed over time, highlighting the immune response development. (A) The graph for HAdV-F40 SFK-immunized sera shows a consistent increase in antibody titers, reaching up to 1:10000 by days 28 and 42. (B) In contrast, the graph for HAdV-F41 SFK-immunized sera shows similar robust responses, with a notable consistency except for a single instance at day 28 where titers plateau at the initial measurement.

HAdV-F40 SFK and HAdV-F41 SFK elicited humoral immune responses, as measured by ELISA. The initial panel, representing the immune sera with HAdV-F40 SFK, demonstrated a sequential increase in immunological response over time, starting from a baseline titer of 1:1000 at day 14, and escalating impressively to 1:10000 by days 28 and 42 post-immunization (Fig. 2A). The second panel, portraying the immune sera with HAdV-F41 SFK, largely mirrors the robust immune response observed in the first panel, with an exception at day 28, where one sample remained at the initial titer of 1:1000 (Fig. 2B). This minor variation does not undermine the overall potent immune response induced by HAdV-F41 SFK. These observations highlight the robust immune responses elicited by our immunization strategy using HAdV-F40 SFK and HAdV-F41 SFK. The consistent antibody responses observed after each immunization underscore their effectiveness. Furthermore, the data suggests the potential of SFK as vaccine candidates for preventing HAdV-F40 and HAdV-F41 infections.

### 3.3 HAdV-F40 SFK and HAdV-F41 SFK induce cross-neutralizing antibody responses determined by MNA

**Fig. 3:**
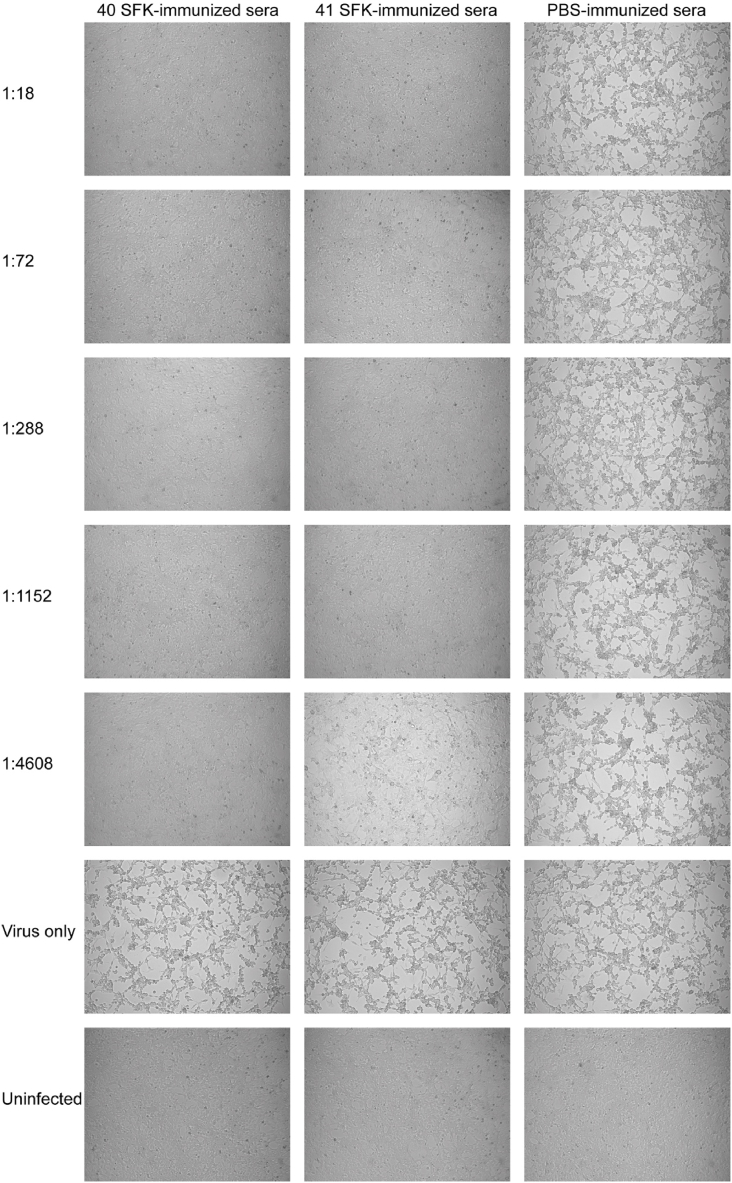
Inhibition of HAdV-F40 growth in HEK-293 cells by immune sera. The series of images compares the CPEs observed in cells treated with serial dilutions of immune sera, ranging from 1:18 to 1:4608. Notably, the immune sera with HAdV-F40 SFK with showed stronger inhibition of HAdV-F40 growth than the immune sera with HAdV-F41 SFK, particularly evident at the highest dilution of 1:4608. The images were captured with a 10 × objective.

**Fig. 4:**
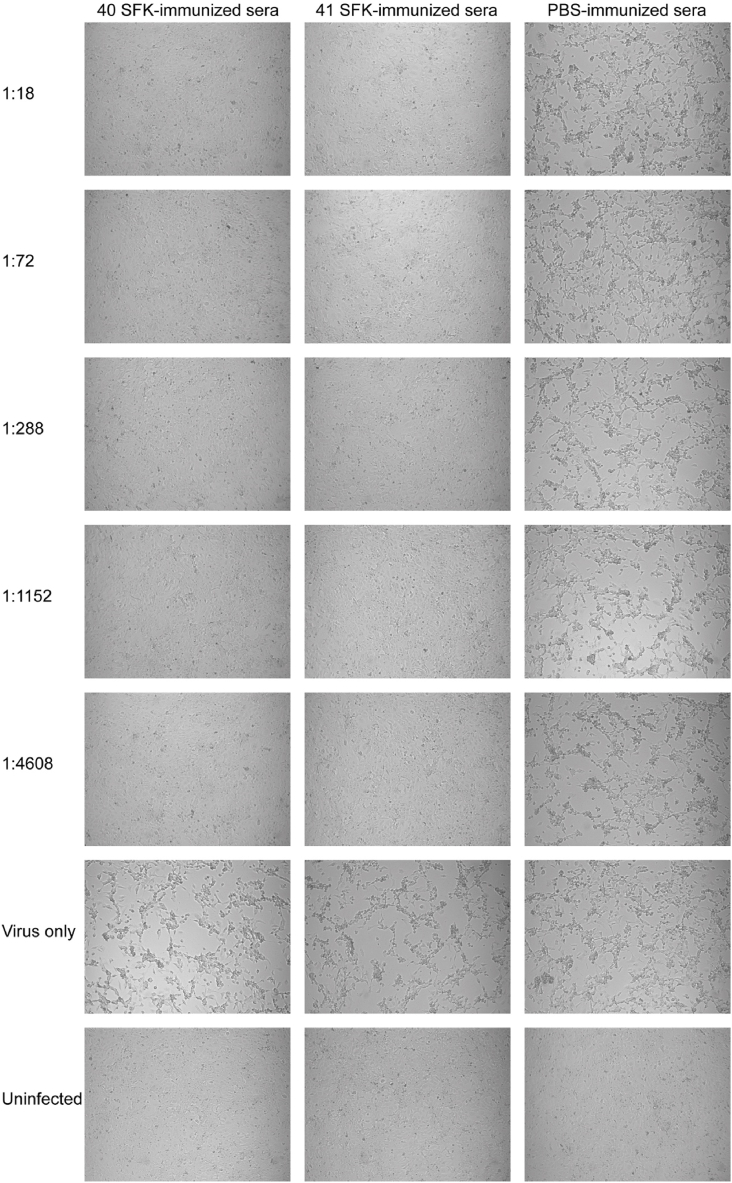
Inhibition of HAdV-F41 growth in HEK-293 cells by immune sera. The series of images compares the CPEs observed in cells treated with serial dilutions of immune sera, ranging from 1:18 to 1:4608. Both immune sera with HAdV-F40 SFK and immune sera with HAdV-F41 SFK exhibited significant cross-neutralizing potential. The images were captured with a 10 × objective.

The HAdV-F40 and HAdV-F41 MNA were conducted on HEK-293 cells using a 96-well microplate. Different dilutions of immune sera were tested to determine neutralization antibody titers against HAdV-F40 and HAdV-F41 and to assess cross-neutralization. The results were inspected under a microscope by the same observer. All five mice in each group exhibited robust immune responses, as reflected by high neutralizing antibody titers. Even at a 4608-fold dilution, adenovirus growth was inhibited by 50 % CPEs. This demonstrated that the immune sera with HAdV-F40 SFK harbored a neutralizing antibody titer of 1:4608 against HAdV-F40 (Fig. 3). Comparable results were obtained for the neutralizing antibody titers against HAdV-F41. Comparable observations were made for the immune sera with HAdV-F41 SFK (Fig. 4). The neutralizing antibody potency against HAdV-F41 in mice inoculated with HAdV-F41 SFK was 1:4608. On the other hand, the neutralizing antibody potency against HAdV-F40 is the same as 1:4608.

### 3.4 HAdV-F40 SFK and HAdV-F41 SFK induce cross-neutralizing antibody responses determined by IFA

**Fig. 5:**
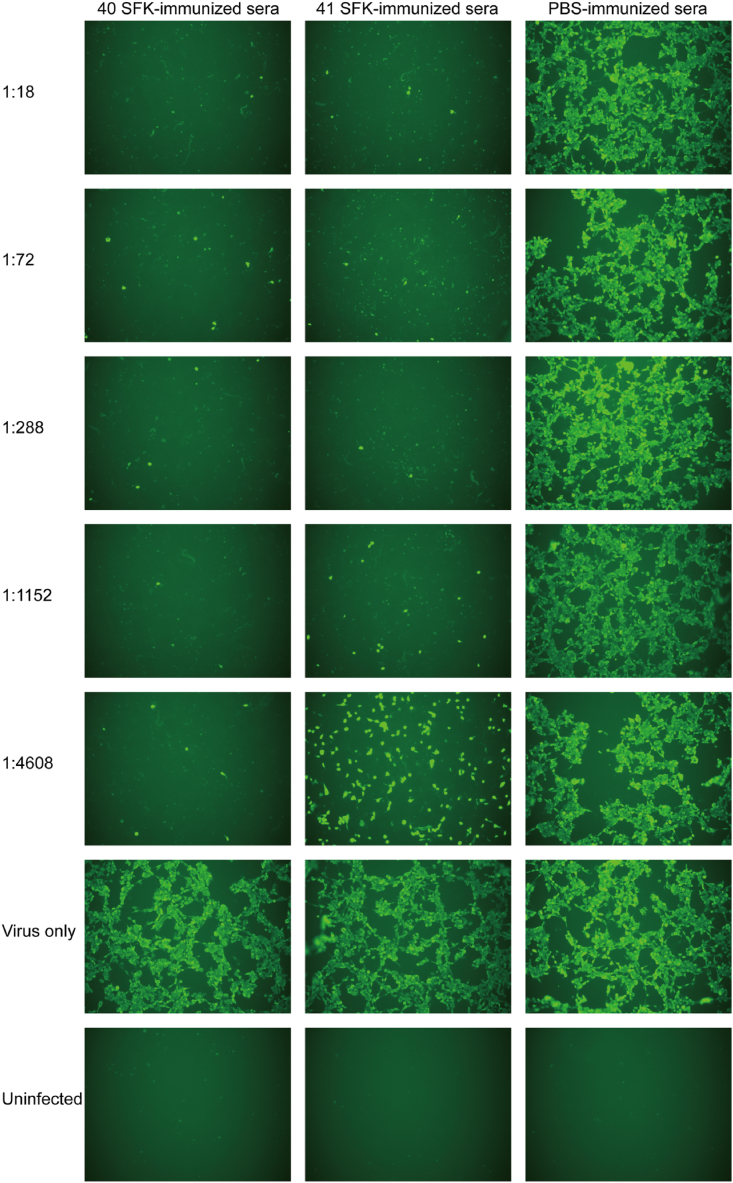
Immunofluorescence analysis of HAdV-F40 response to immune sera. Fluorescence imaging of cells treated with HAdV-F40 SFK and HAdV-F41 SFK immune sera ranging from 1:18 to 1:4608, displaying the response to HAdV-F40. The images were captured with a 10 × objective.

**Fig. 6:**
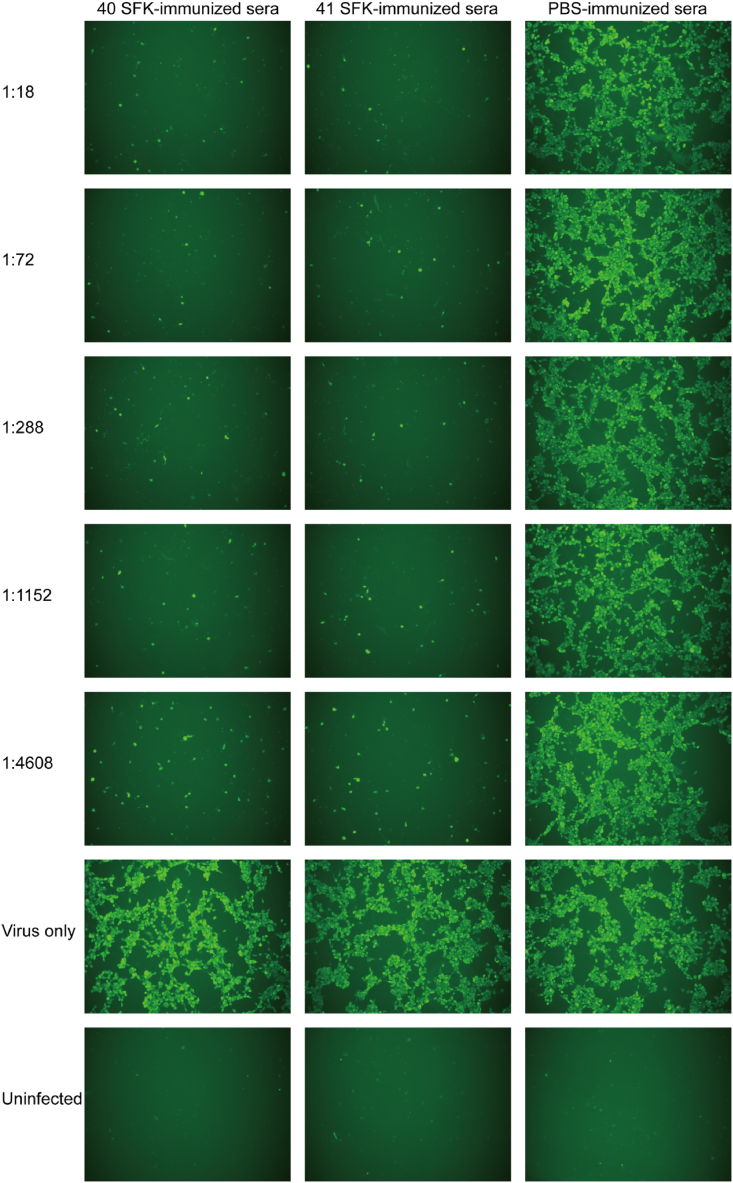
Immunofluorescence analysis of HAdV-F41 response to immune sera. Fluorescence imaging of cells treated with HAdV-F40 SFK and HAdV-F41 SFK immune sera ranging from 1:18 to 1:4608, demonstrating the response to HAdV-F41. The images were captured with a 10 × objective.

Following infection with HAdV-F40 and HAdV-F41 respectively, the cells displayed green fluorescence as a result of the interaction between the primary and FITC-labeled secondary antibodies. The intensity of this green fluorescence directly correlated with HAdVs infection levels and serum neutralization efficiency. Inhibition of HAdV-F40 growth in HEK-293 cells by immune sera with HAdV-F40 SFK and immune sera with HAdV-F41 SFK, visualized through immunofluorescence. Cells treated with serially diluted immune sera (from 1:18 to 1:4608) show absence of green fluorescence, indicating effective neutralization of HAdV-F41. The dilution (1:1152) of both immune sera with HAdV-F40 SFK and immune sera with HAdV-F41 SFK demonstrates almost complete inhibition of viral cytopathic effects, but the differences appear at the highest dilution 1:4608. In contrast, cells treated with PBS-immunized serum exhibit intense green fluorescence similar to the 'Virus only' control, highlighting active viral infection (Fig. 5). Inhibition of HAdV-F41 growth in HEK-293 cells by immune sera with HAdV-F40 SFK and immune sera with HAdV-F41 SFK, assessed through immunofluorescence. Immune sera with HAdV-F40 SFK and immune sera with HAdV-F41 SFK at all dilutions from 1:18 to 1:4608 show absence of green fluorescence, indicating effective neutralization across all tested concentrations. This consistent lack of fluorescence demonstrates the potent neutralizing ability of both immune sera with HAdV-F40 SFK and immune sera with HAdV-F41 SFK against HAdV-F41. Contrastingly, cells treated with PBS-immunized sera display intense green fluorescence, akin to the 'Virus only' control, clearly indicating unmitigated viral infection (Fig. 6).

## 4 Discussion

HAdV-F40 and HAdV-F41 are strongly associated with diarrheal diseases, which are notable causes of child mortality in developing countries [28]. Previous studies have reported a high occurrence of enteric adenoviruses in children suffering from diarrhea compared to their healthy counterparts [10,29,30]. Therefore, understanding species F adenoviruses becomes imperative in developing strategies against diarrheal diseases and improving child health in resource-limited areas.

Our investigation offers a comprehensive look into the cross-reactivity and neutralization capabilities of immune sera with HAdV-F40 SFK and immune sera with HAdV-F41 SFK. Notably, the neutralizing antibody titers for HAdV-F40 and HAdV-F41 were comparably high at 1:4608. Consistent with the findings that these viruses share almost identical amino acid sequences [31], immune sera with HAdV-F40 SFK showed pronounced neutralizing activity against HAdV-F41 and vice versa, confirming the potential for cross-neutralizing antibody-based treatments. However, immune sera with HAdV-F40 SFK demonstrated a distinct advantage in our experimental settings. It is noteworthy that while immune sera with HAdV-F41 SFK effectively inhibited a significant portion of the CPEs, they were not as potent as the immune sera with HAdV-F40 SFK in neutralizing HAdV-F41. This observation suggests a potential superior protective effect of the HAdV-F40 SFK over HAdV-F41 SFK. This distinct advantage of anti-HAdV-F40 sera underscores its potential superior protective effect, which is consistent across all five mice, as detailed further in this study.

In light of these observations, the potential of species F adenoviruses as gene delivery vectors targeting the gastrointestinal tract [32] suggests promising avenues for subsequent research. These investigations could concentrate on the propagation of these adenoviruses and the rigorous analysis of their induced neutralizing antibody profiles. Traditionally, various adenovirus vectors were employed given the minimal cross-reactivity among NAbs against different adenovirus species, thus aiding in circumventing pre-existing NAbs. However, consistent with earlier findings [[33], [34], [35]], our results confirm the cross-reactivity between the NAbs of HAdV-F40 and HAdV-F41. It has been documented that polyclonal sera against these types can cross-neutralize, suggesting that the production of NAbs against HAdV-F40 in a host's immune system might also neutralize HAdV-F41, regardless of previous exposure to HAdV-F41. Notably, the mechanism of adenovirus neutralization by antibodies, particularly those targeting the exposed hexon loops, is critical for understanding the broad reactivity observed [[36], [37], [38], [39]]. These epitopes are significant as they facilitate the broad neutralization capacities of the antibodies. Additionally, considering the *in vivo* efficacy of anti-fiber antibodies, it is crucial to ensure that antibody concentrations achieved in clinical settings are sufficient for neutralization. A study on HAdV-B55 demonstrated that while high doses of fiber-targeting antibodies are necessary to prevent infection, hexon-targeting antibodies can be effective at significantly lower doses, suggesting variability in the effective concentrations needed for different antibody types [40]. Additionally, the role of intracellular mechanisms, such as those mediated by tripartite motif-containing 21 where antibodies target the internalized virions for degradation, should be considered in the context of adenovirus neutralization [41].

Furthermore, considering that infection of 293 cells is mediated primarily by LFK binding to coxsackie and adenovirus receptor, it raises intriguing questions about the mechanism by which antibodies targeting SFK can neutralize the virus. According to a previous study, it is crucial to explore whether these antibodies contribute to neutralization primarily through intracellular pathways rather than by preventing virion attachment at neutral pH [42]. This understanding might reveal additional strategies by which adenovirus infections could be targeted therapeutically, particularly in leveraging the specific interactions between adenoviruses and host cells.

Drawing from our in vitro insights, plans for subsequent research will transition towards *in vivo* experimentation. The observed potential of immune sera with HAdV-F40 SFK and immune sera with HAdV-F41 SFK suggests further evaluation in suitable animal models. This work provides a foundation for developing a subunit vaccine targeting both HAdV-F40 and HAdV-F41. It underscores the efficacy of SFK immunization in inducing a cross-neutralizing antibody response and offers insights into the protective capabilities of neutralizing antibodies against HAdV-F40 and HAdV-F41. Subsequent research will aim to optimize the immunogenicity and efficacy of this subunit vaccine, contributing to strategies against diseases associated with HAdV-F40 and HAdV-F41.

## Funding

This study was sponsored by the 10.13039/501100001809National Natural Science Foundation of China (82072264), the grant of the 10.13039/100013262State Key Laboratory of Respiratory Disease, Guangdong-Hong Kong-Macao Joint Laboratory of Respiratory Infectious Disease (GHMJLRID-Z-202104), 10.13039/501100003453Natural Science Foundation of Guangdong Province, China (2021A1515110550, 2021A1515220043, and 2023A1515010589) and Undergraduate Science and Technology Innovation Training Program (2022A003).

## Ethics statement

All animal experiments were approved by the Laboratory Animal Ethic Committee of Affiliated First Hospital of Guangzhou Medical University, and performed in accordance with the guidelines of the Animal Care and Use Committee of Affiliated First Hospital of Guangzhou Medical University.

## Data availability statement

The data are available from the corresponding authors when required.

## CRediT authorship contribution statement

**Zhenwei Liu:** Writing – review & editing, Resources, Project administration, Methodology, Funding acquisition, Data curation. **Guolu Tang:** Writing – review & editing, Writing – original draft. **Yinghui Peng:** Software, Resources. **Jixian Lan:** Investigation, Conceptualization. **Yuting Xian:** Validation, Project administration, Conceptualization. **Xingui Tian:** Supervision, Methodology, Funding acquisition. **Dehui Chen:** Supervision, Funding acquisition.

## Declaration of competing interest

The authors declare that they have no known competing financial interests or personal relationships that could have appeared to influence the work reported in this paper.
