# Supplementary material for: The short fiber knobs of human adenovirus in species F elicit cross-neutralizing antibody responses
Source: Heliyon. 2024 Aug 3;10(15):e35783. doi: 10.1016/j.heliyon.2024.e35783 (PMC11337035; doi:10.1016/j.heliyon.2024.e35783)
Supplement: Multimedia component 1 [file mmc1.docx]

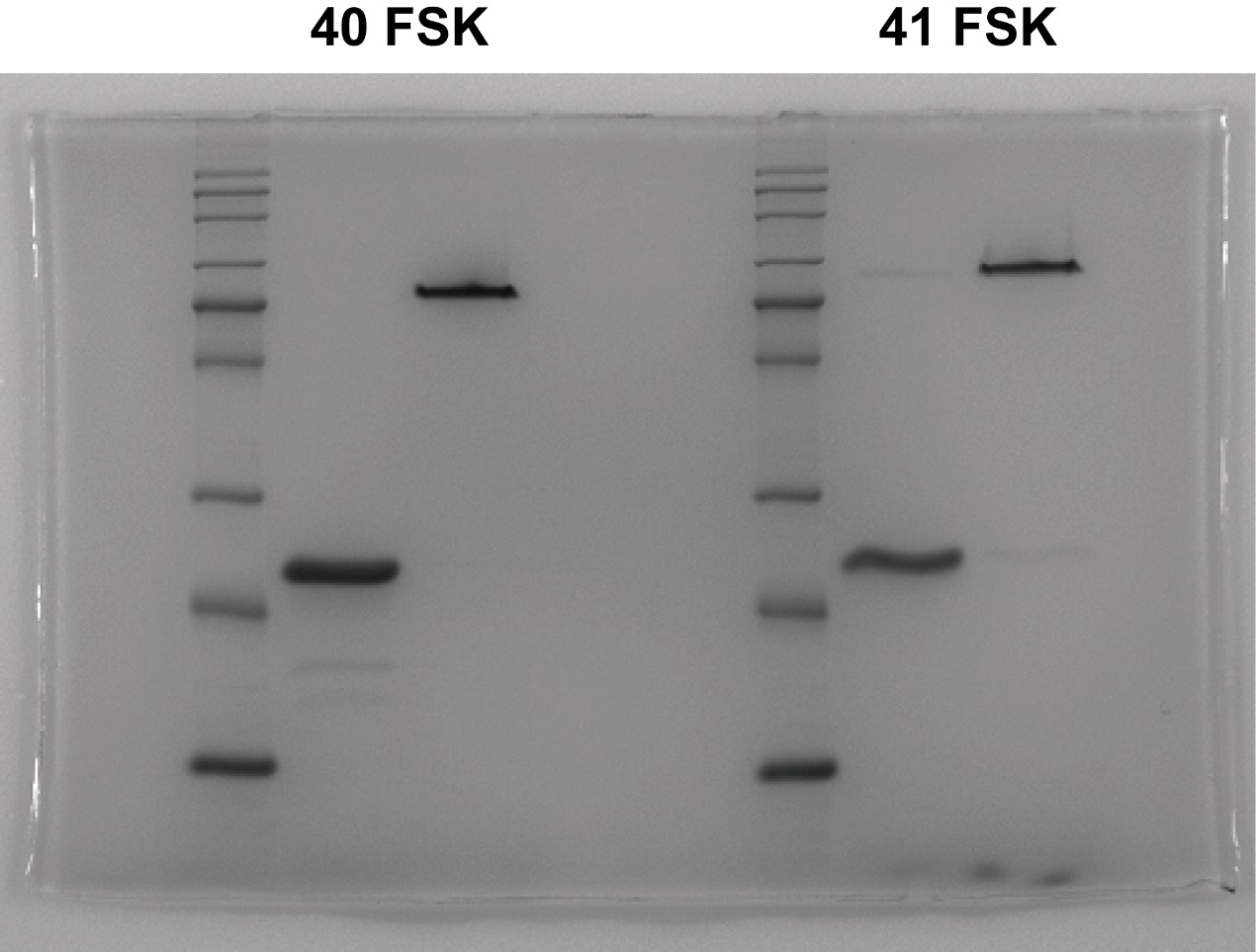


Fig. S1. The full, non-adjusted image of SDS-PAGE results of purified HAdV-F40 SFK and purified HAdV-F41 SFK.
